# Construction and validation of a machine learning-based prediction model for 48-hour reintubation risk in mechanically ventilated patients

**DOI:** 10.3389/fmed.2026.1788254

**Published:** 2026-03-19

**Authors:** Wei Zhang, Xing Wei Di

**Affiliations:** 1 School of Nursing, Jinzhou Medical University, Jinzhou, Liaoning, China; 2 Department of Critical Care Medicine, The First Affiliated Hospital of Jinzhou Medical University, Jinzhou, Liaoning, China

**Keywords:** extubation failure, machine learning, mechanical ventilation, reintubation, risk prediction model

## Abstract

**Background:**

In the ICU, reintubation after extubation in mechanically ventilated patients is often followed by adverse clinical events and is associated with longer ICU and hospital stays as well as increased mortality. Therefore, timely and accurate assessment of reintubation risk is clinically important for supporting extubation decisions and early management. Although several scoring systems and prediction models have been proposed, machine learning approaches may offer additional value by integrating multidimensional clinical information and potentially improving predictive performance.

**Methods:**

This retrospective observational study included mechanically ventilated patients admitted to the intensive care unit (ICU) of the First Affiliated Hospital of Jinzhou Medical University between January 2022 and October 2025. Patients were randomly allocated at a 7:3 ratio to a training set (*n* = 496) and a test set (*n* = 211). In the training set, random forest-recursive feature elimination (RF-RFE) and the least absolute shrinkage and selection operator (LASSO) were used for feature selection, and prediction models were developed using seven machine learning algorithms. Model performance in the test set was evaluated by discrimination [area under the receiver operating characteristic curve (AUROC)], calibration (calibration curve), and clinical utility [decision curve analysis (DCA)] to identify the best-performing model. The final prediction tool was presented as both a static nomogram and a web-based dynamic nomogram.

**Results:**

A total of 707 mechanically ventilated patients were included, and the 48-h reintubation rate was 17.39% (123/707). Compared with the other six models, the LASSO-logistic regression (LASSO-LR) model achieved superior discrimination in the test set (AUROC = 0.879, 95% CI 0.814–0.935) and showed the best calibration (Brier score = 0.090, 95% CI 0.063–0.119). DCA indicated that this model provided a measurable net clinical benefit for predicting reintubation within 48 h after extubation among mechanically ventilated patients. Accordingly, LASSO-LR was selected as the optimal model and further implemented as a general static nomogram and a web-based dynamic nomogram (https://predict-for-reintubation-within-48-hours.shinyapps.io/dynnomapp/).

**Conclusion:**

We developed and compared seven models for predicting reintubation risk after extubation in mechanically ventilated patients, among which the LASSO-LR model demonstrated the best overall performance. Visualizing the model as static and dynamic nomograms that integrate key predictors may facilitate early identification of patients at high risk of reintubation and support targeted preventive and management strategies in clinical practice.

## 1 Introduction

Invasive mechanical ventilation (IMV) remains one of the most widely used life-support interventions in critical care. Approximately 20–40% of patients admitted to intensive care units (ICUs) require mechanical ventilation to maintain adequate oxygenation and ventilation (1). However, prolonged ventilation is associated with higher risks of ventilator-associated pneumonia, ventilator-induced lung injury, diaphragmatic dysfunction, and ventilator dependence (2, 3). Accordingly, once the underlying condition is controlled and respiratory function improves, timely liberation from the ventilator and extubation are essential to reduce ventilation-related complications. Nevertheless, extubation decisions continue to represent a major challenge in ventilatory management of critically ill patients (4). Both unnecessary prolongation of intubation and premature extubation can lead to adverse outcomes and may increase mortality risk (5, 6).

To improve the likelihood of successful extubation, clinicians commonly rely on indicators such as the rapid shallow breathing index (RSBI) and spontaneous breathing trials (SBTs). RSBI was originally proposed by Yang and Tobin (7) and is defined as the ratio of respiratory frequency to tidal volume (f/VT) measured during a 1-min spontaneous breathing assessment. In practice, RSBI may be influenced by endotracheal suctioning, smaller endotracheal tube diameter, and multiple factors that alter respiratory rate (8). Moreover, evidence suggests that RSBI alone has limited predictive value for extubation success, and its optimal threshold varies substantially across populations; thus, it is often interpreted in conjunction with other parameters to improve performance (9). SBT is widely regarded as the reference standard for evaluating readiness to wean (10), typically by observing arterial blood gases and hemodynamic changes after discontinuation of positive-pressure ventilation. However, due to cardiopulmonary interactions under positive pressure, patients with impaired cardiac function may develop heart failure after weaning, while others may experience respiratory muscle fatigue; during the trial, dynamic cardiac monitoring can also be relatively complex in routine practice (11). Even among patients who pass an SBT and undergo planned extubation, approximately 10–20% of adult patients experience extubation failure and require reintubation within 48 h following extubation (9).

Reintubation following extubation failure is associated with a spectrum of unfavorable outcomes, including prolonged duration of mechanical ventilation, increased risk of ventilator-associated pneumonia, longer ICU and hospital length of stay, higher hospitalization costs, increased in-hospital mortality, and a greater likelihood of tracheostomy (12, 13). Compared with patients who are successfully extubated, those who fail extubation have been reported to have a 7-fold higher risk of death and a 31-fold longer ICU length of stay (14). These consequences not only intensify patient suffering and financial burden but also consume substantial healthcare resources. Therefore, developing a simple, generalizable risk-prediction model that supports individualized assessment to assist extubation decision-making is of clear clinical importance.

In recent years, with the rapid expansion of machine learning (ML) in healthcare, clinical prediction models built from electronic health records (EHRs) or large clinical databases for prognostic assessment have become a major research focus. Traditional statistical approaches primarily emphasize associations between variables, whereas ML methods are better suited to high-dimensional and heterogeneous data, and may capture non-linear patterns and potential interactions, thereby offering the prospect of improved predictive accuracy (15). Although a large body of work has examined mechanical ventilation, much of it has focused on identifying risk factors (10, 16, 17), and relatively few studies have developed risk-prediction models specifically for reintubation within 48 h after extubation.

Against this background, constructing prediction models based on routinely available and clinically accessible variables—while balancing accuracy with practicality—could enable clinicians to make timelier and more appropriate extubation decisions and improve ICU resource allocation. In this study, we developed models using multiple ML algorithms and selected the best-performing approach to build an online prediction system.

## 2 Materials and methods

### 2.1 Patient selection

This retrospective study analyzed clinical data from mechanically ventilated patients admitted to the ICU of the First Affiliated Hospital of Jinzhou Medical University between January 2022 and October 2025. A total of 707 patients were included, of whom 123 underwent reintubation and 584 were successfully extubated. Using R software, all patients were randomly assigned in a 7:3 ratio to a training set (*n* = 496) and a test set (*n* = 211). The training set was used for model development, and the test set was used for internal validation. This study was approved by the Ethics Committee of Jinzhou Medical University (Approval No. JZMULL2025340).

### 2.2 Subjects of the study

Inclusion criteria were: (1) age ≥18 years; (2) endotracheal intubation; and (3) duration of ICU mechanical ventilation ≥24 h. Exclusion criteria were: (1) unplanned extubation; and (2) missing baseline information ≥20%.

### 2.3 Outcomes and diagnostic criteria

The primary outcome was reintubation within 48 h after extubation. Secondary outcomes included hospital length of stay, ICU length of stay, and in-hospital mortality.

### 2.4 Data collection

Based on literature review, clinical interpretability, and data availability, we collected and analyzed candidate predictors, including sex, age, chronic obstructive pulmonary disease, shock, pneumonia, acute respiratory distress syndrome, respiratory failure, coronary artery disease, heart failure, sepsis, trauma, severity scores within 24 h of ICU admission (APACHE II, SOFA, and GCS), vital signs within 30 min before extubation (respiratory rate and heart rate), laboratory results within 24 h before extubation (oxygenation index, arterial oxygen saturation, partial pressure of arterial oxygen, partial pressure of arterial carbon dioxide, hemoglobin, albumin, B-type natriuretic peptide, and pH), ventilator parameters (positive end-expiratory pressure, tidal volume, and minute ventilation), abundant secretions, number of SBT attempts (the SBT was performed using pressure support ventilation, with PEEP set at 5–8 cmH₂O and a duration of 30–120 min), duration of mechanical ventilation before extubation and RSBI (measured during the SBT prior to extubation). All predictor variables were defined using information available before the extubation decision. No post-extubation physiological parameters, treatment variables, or outcome-related data were incorporated into model training, thereby minimizing the risk of data leakage.

### 2.5 Statistical analysis

Data processing and statistical analyses were performed using Python (v3.10.1) and R (v4.5.1). Variables with >20% missingness were excluded. For the remaining variables, missing values were handled using multiple imputation with the *mice* package in R. Normality of continuous variables was assessed using the Shapiro–Wilk test. Normally distributed continuous variables are presented as mean ± standard deviation (SD) and were compared using the Student’s *t* test. Non-normally distributed variables are reported as median (interquartile range) [M (Q1, Q3)] and were compared using the Mann–Whitney *U* test. Categorical variables are expressed as counts (percentages) and were compared using the *χ*^2^ test.

To mitigate overfitting, feature selection was performed using LASSO with 10-fold cross-validation, in combination with RF-RFE. The intersection of features selected by both methods was retained for model development. The dataset was randomly split in a 7:3 ratio into a training set and a test set. Given the class imbalance in the training set, the synthetic minority over-sampling technique (SMOTE) was applied to rebalance outcome classes (18). During model training, 5-fold cross-validation coupled with grid search was used to identify optimal hyperparameters. Based on the selected predictors, seven models were developed: LASSO-LR, decision tree (DT), artificial neural network (ANN), light gradient boosting machine (LightGBM), random forest (RF), extreme gradient boosting (XGBoost), and support vector machine (SVM).

Model performance was evaluated in the test set using accuracy, precision, sensitivity, specificity, *F*_1_-score, positive predictive value, negative predictive value, and AUROC, and the best-performing model was selected accordingly. Calibration was further examined using calibration curves and the Hosmer–Lemeshow goodness-of-fit test to assess agreement between predicted and observed risks in the nomogram. Finally, the optimal model was visualized as a static nomogram and implemented as a web-based dynamic nomogram. All tests were two-sided, with a significance level set at *α* = 0.05.

## 3 Results

### 3.1 Baseline characteristics of the patients

**Figure 1:**
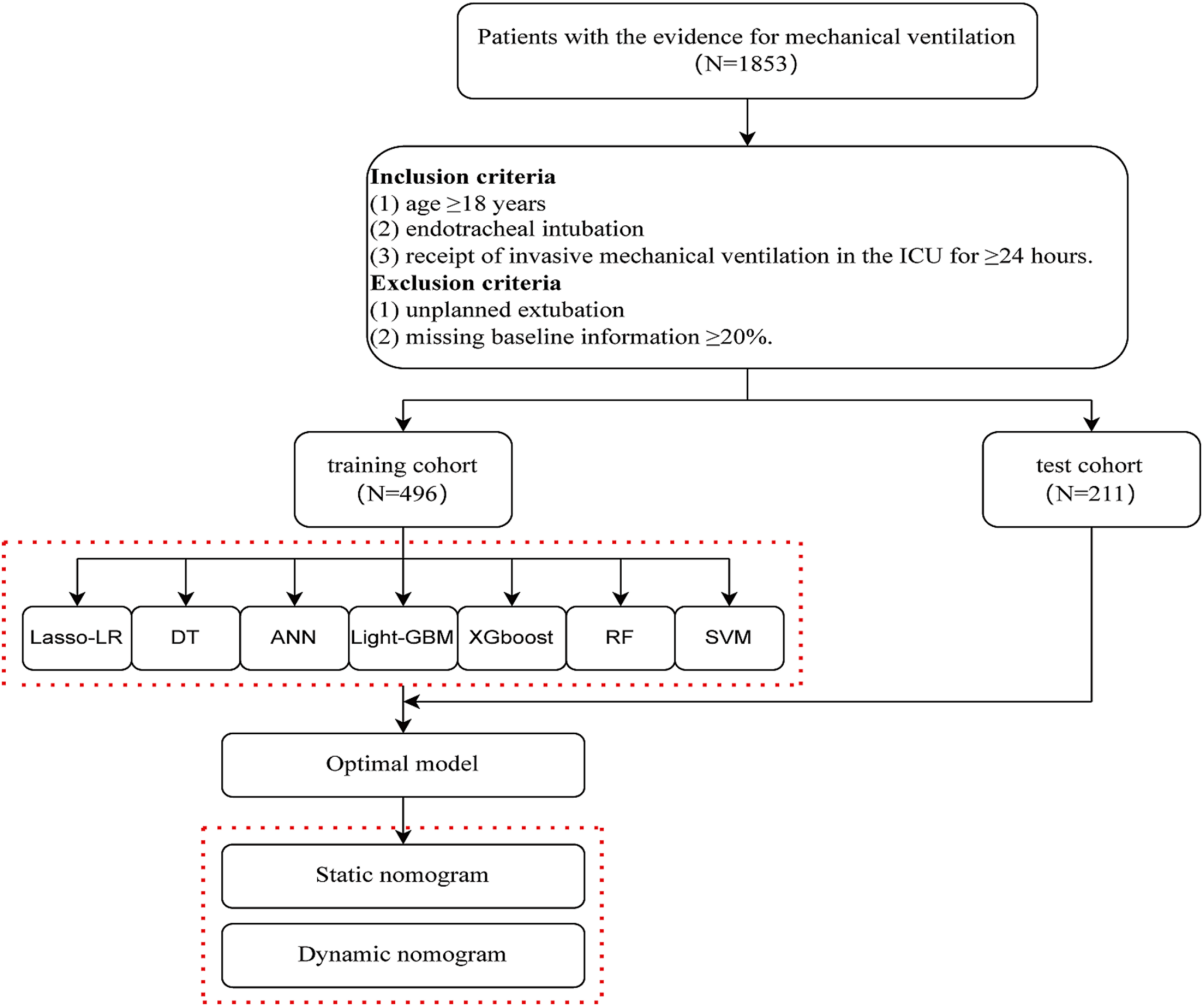
Flow chart of the study.

According to the predefined inclusion and exclusion criteria, 707 intubated patients were included and allocated to the training cohort (*n* = 496) and the test cohort (*n* = 211) (Figure 1).

Baseline characteristics are summarized in Table 1. Overall, 123 patients required reintubation, whereas 584 were successfully extubated, yielding a reintubation rate of 17.39%. Significant between-group differences were observed across multiple variables, including in-hospital mortality; comorbidities/complications (sepsis and pneumonia); airway secretions; age; ICU length of stay; GCS score; APACHE II and SOFA scores; albumin; oxygenation index; pH; PaO₂; arterial oxygen saturation; heart rate; respiratory rate; tidal volume; number of SBT attempts; RSBI; and duration of mechanical ventilation before extubation (all *p* < 0.05).

**Table 1:** The characteristics of the whole cohort.

Variable	Total (*n* = 707)	Not reintubation (*n* = 584)	Reintubation (*n* = 123)	*p*
Age (years)	63.00 [51.00, 72.0]	62.00 [50.00, 71.0]	67.00 [56.50, 74.00]	0.002
Gender, *N* (%)				
Female	457 (64.64)	376 (64.38)	81 (65.85)	0.837
Male	250 (35.36)	208 (35.62)	42 (34.15)	
COPD, *N* (%)	31 (4.38)	23 (3.94)	8 (6.50)	0.307
Trauma, *N* (%)	119 (16.83)	95 (16.27)	24 (19.51)	0.458
Shock, *N* (%)	144 (20.37)	111 (19.01)	33 (26.83)	0.067
ARDS, *N* (%)	71 (10.04)	53 (9.08)	18 (14.63)	0.089
Respiratory failure, *N* (%)	295 (41.73)	237 (40.58)	58 (47.15)	0.214
Hypertension, *N* (%)	204 (28.85)	165 (28.25)	39 (31.71)	0.51
CAD, *N* (%)	87 (12.31)	75 (12.84)	12 (9.76)	0.426
Heart failure, *N* (%)	82 (11.60)	70 (11.99)	12 (9.76)	0.584
Neurologic diagnosis, *N* (%)	330 (46.68)	263 (45.03)	67 (54.47)	0.071
Sepsis, *N* (%)	96 (13.58)	70 (11.99)	26 (21.14)	0.011
Pneumonia, *N* (%)	445 (62.94)	345 (59.08)	100 (81.30)	<0.001
Abundant secretions, *N* (%)	368 (52.05)	278 (47.60)	90 (73.17)	<0.001
Death, *N* (%)	44 (6.22)	11 (1.88)	33 (26.83)	<0.001
Hospital stay (hours), median (IQR)	381.15 [207.88, 600.00]	360.00 [192.00, 611.41]	408.00 [285.29, 552.00]	0.103
ICU_stay (hours), median (IQR)	170.00 [108.76, 269.52]	166.20 [96.00, 240.00]	312.00 [192.00, 432.00]	<0.001
GCS score, median (IQR)	9.00 [5.00, 14.00]	11.00 [6.00, 15.00]	5.00 [3.00, 9.00]	<0.001
APACHE II score, median (IQR)	18.00 [13.00, 23.00]	17.00 [12.00, 21.00]	27.00 [23.00, 29.00]	<0.001
SOFA score, median (IQR)	6.00 [4.00, 8.00]	5.00 [3.00, 7.00]	8.00 [7.00, 10.00]	<0.001
BNP (ng/L), median (IQR)	87.00 [32.20, 245.50]	81.00 [31.28, 241.07]	107.00 [42.90, 265.25]	0.066
Hemoglobin (g/L), median (IQR)	100.00 [86.90, 117.00]	100.00 [87.90, 118.00]	100.00 [83.00, 115.20]	0.365
Albumin (g/L), median (IQR)	31.80 [29.23, 35.00]	32.00 [29.99, 35.00]	30.76 [28.60, 33.91]	0.012
PaO_2_/FiO_2_ (mmHg), median (IQR)	305.00 [246.15, 367.30]	308.60 [247.30, 374.30]	297.10 [245.20, 338.30]	0.012
PH, median (IQR)	7.47 [7.43, 7.50]	7.47 [7.42, 7.50]	7.47 [7.43, 7.52]	0.036
PaCO_2_ (mmHg), median (IQR)	37.00 [32.70, 40.85]	37.00 [32.70, 40.92]	36.60 [32.80, 40.20]	0.697
PaO_2_ (mmHg), median (IQR)	115.00 [97.95, 139.00]	116.00 [98.83, 140.25]	112.00 [93.05, 135.00]	0.046
SaO_2_ (%), median (IQR)	99.00 [98.00, 99.50]	99.00 [98.00, 99.60]	98.80 [98.00, 99.00]	<0.001
HR (rate/min), median (IQR)	90.00 [78.00, 102.00]	89.00 [77.75, 100.00]	96.00 [81.00, 109.50]	<0.001
RR (rate/min), median (IQR)	21.00 [19.00, 23.00]	20.00 [19.00, 22.00]	23.00 [20.00, 28.00]	<0.001
Tidal volume (mL/kg), median (IQR)	385.00 [328.00, 457.52]	390.26 [337.29, 460.00]	349.50 [295.90, 425.71]	<0.001
PEEP (cmH_2_O), median (IQR)	6.00 [5.00, 6.00]	6.00 [5.00, 6.00]	6.00 [5.00, 6.50]	0.273
Minute volume (L/min), median (IQR)	8.05 [7.10, 9.20]	8.00 [7.11, 9.10]	8.20 [7.00, 9.85]	0.424
Number of SBTs, median (IQR)	1.00 [1.00, 2.00]	1.00 [1.00, 1.00]	2.00 [1.00, 3.00]	<0.001
RSBI (breaths/min/L), median (IQR)	52.50 [42.47, 70.07]	51.28 [41.54, 65.79]	65.10 [51.00, 97.38]	<0.001
Duration of MV (hours), median (IQR)	137.00 [72.00, 216.00]	120.00 [59.50, 192.00]	240.00 [168.00, 344.00]	<0.001

### 3.2 Feature selection

#### 3.2.1 Multicollinearity

**Figure 2:**
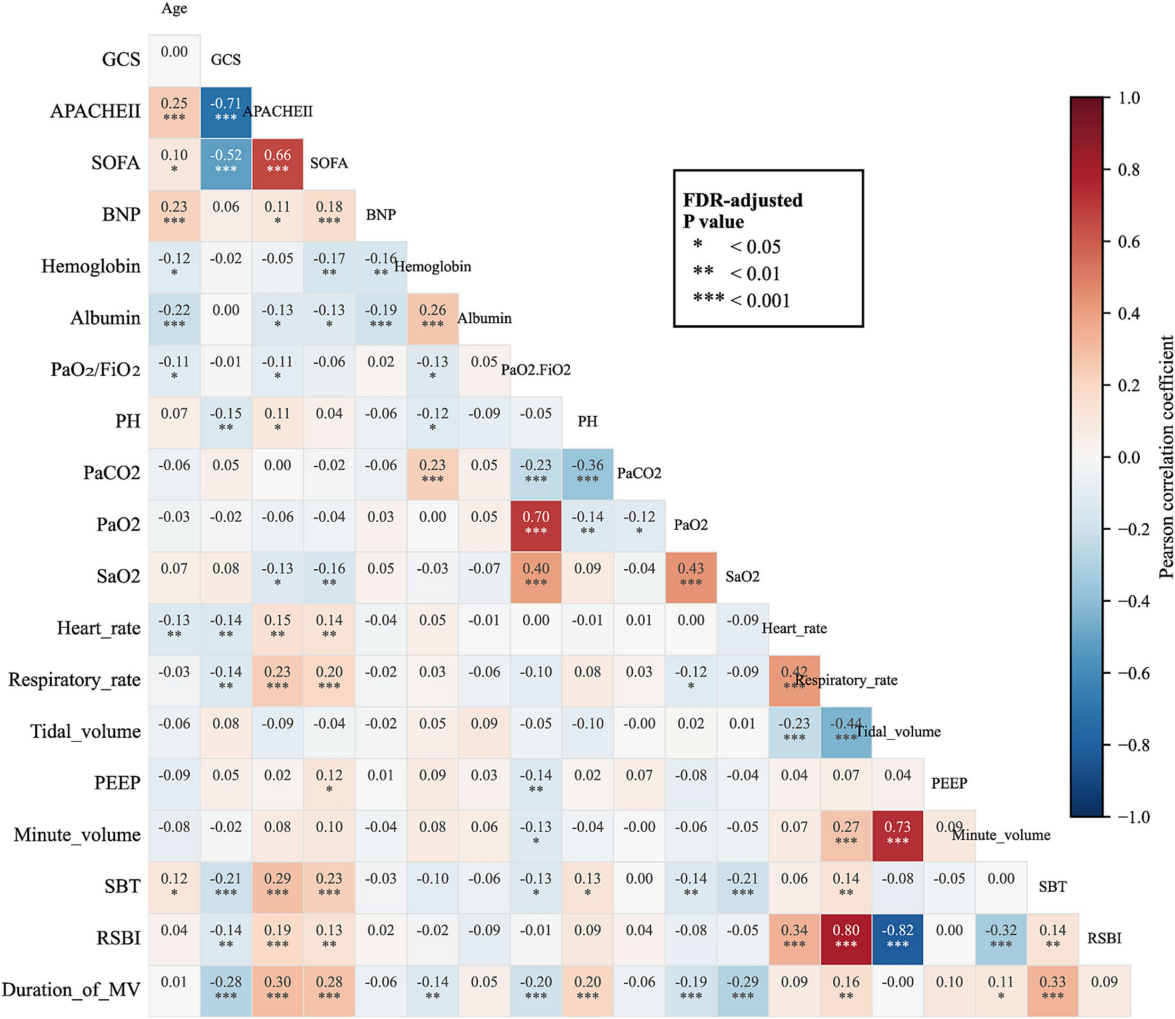
Pearson correlation heatmap of candidate variables in the training cohort.

Strong correlations among predictors can undermine model interpretability and compromise the identification of statistically meaningful variables. To assess the risk of multicollinearity, we first calculated Pearson correlation coefficients for all candidate predictors and visualized them using a correlation heatmap (Figure 2) to identify highly correlated variable pairs or clusters. A threshold of *r* = 0.7 was applied: when (|*r*| ≥ 0.7) and the FDR-adjusted *p*-value was <0.05, one variable from the correlated pair was removed. Ultimately, tidal volume and the GCS score were excluded.

#### 3.2.2 RF-RFE algorithm

**Figure 3:**
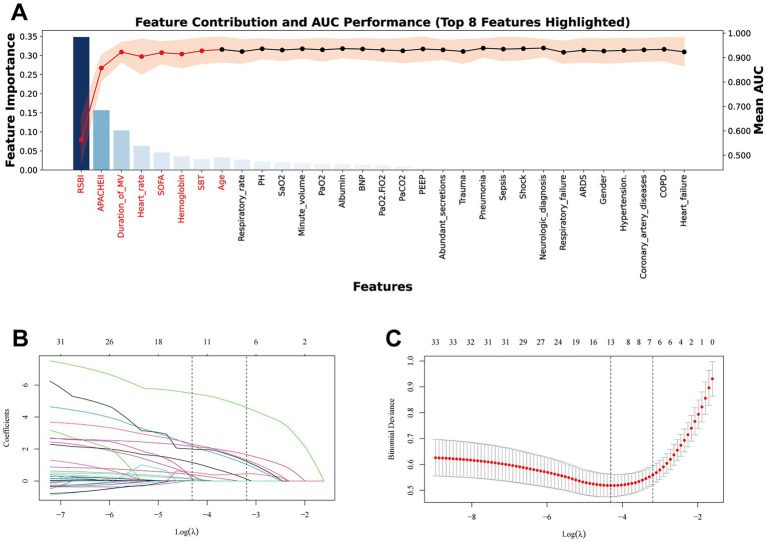
Selection of clinical features. **(A)** Recursive elimination of random forest features. **(B)** Coefficient profiles of candidate predictors as a function of the regularization parameter (log *λ*). With increasing *λ*, most coefficients shrink toward zero, and only variables with substantial predictive contribution remain. **(C)** Ten-fold cross-validation for tuning parameter selection in the LASSO model. The left dashed line represents the minimum cross-validated error (*λ*_min), and the right dashed line indicates the 1-SE criterion (*λ*_1SE) used for the final model selection.

After redundancy reduction, we applied RF-RFE to iteratively remove features with lower contributions, enabling feature selection to proceed in tandem with model training. At each iteration, feature importance was recalculated, which better accounts for inter-feature dependencies and helps reduce information loss during the selection process (19). The stopping criterion was the best-performing area under the AUC. Because model performance did not improve after the 8th iteration (Figure 3A), the preliminary set of predictors retained by RF-RFE included RSBI, APACHE II, duration of mechanical ventilation, heart rate, SOFA score, hemoglobin, number of SBT attempts, and age.

#### 3.2.3 LASSO regression

LASSO regression was further used for feature selection with 10-fold cross-validation. Figures 3B,C present the LASSO coefficient path and the cross-validation curve. As the penalty parameter (*λ*) increased, most coefficients gradually shrank to zero, leaving only predictors with relatively higher predictive weights. Using the 1-SE rule, the non-zero coefficients retained were RSBI, APACHE II, duration of mechanical ventilation, heart rate, SOFA score, respiratory rate, and number of SBT attempts. Taking the intersection of predictors selected by LASSO and RF-RFE, the final model included RSBI, APACHE II, duration of mechanical ventilation, heart rate, SOFA score, and number of SBT attempts.

### 3.3 Model development

Based on the six predictors identified above, we constructed seven prediction models using machine-learning algorithms that have been widely applied and have shown robust performance in prior studies (18–20): LASSO-LR, DT, ANN, LightGBM, RF, XGBoost, and SVM. Because reintubation after extubation is a relatively low-incidence event among mechanically ventilated patients, the training set was subject to class imbalance. To mitigate this issue, we applied the SMOTE to improve minority-class recognition while reducing the risk of overfitting (21).

During model training, hyperparameters were tuned using grid search with 5-fold cross-validation. The hyperparameter set yielding the highest area under the AUROC was selected for subsequent evaluation in the test set. The 95% confidence intervals (CIs) for AUROC were estimated using bootstrap resampling. With the exception of LASSO-LR, the optimal hyperparameters were as follows: DT, {ccp_alpha = 0.0, max_depth = None, max_features = None, min_samples_split = 20}; RF, n_estimators = 350 and max_features = 2; XGBoost, {learning_rate = 0.2, max_depth = 10, n_estimators = 50, subsample = 0.8}; LightGBM, {colsample_bytree = 0.6, learning_rate = 0.2, n_estimators = 100, num_leaves = 31, subsample = 0.6}; SVM, {C = 0.1, degree = 2, gamma = scale, kernel = linear}; and ANN, {activation = logistic, hidden_layer_sizes = (100, 100)}.

### 3.4 Model evaluation and selection

**Figure 4:**
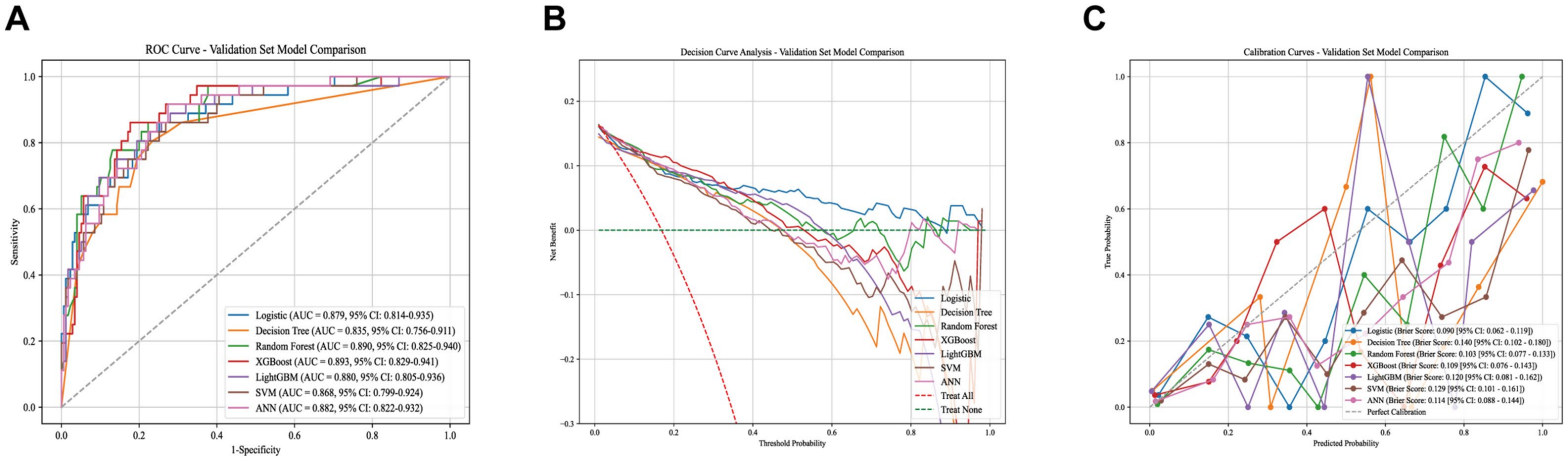
Summary plot of machine learning performance evaluation (test set). **(A)** ROC curve. **(B)** DCA curve. **(C)** Calibration plot.

We compared the generalizability of the seven models in the test set. Overall, the logistic regression-based model demonstrated the best performance. In the test cohort, it achieved an AUROC of 0.879 (95% CI, 0.814–0.935). Importantly, while its discrimination was comparable to that of the ensemble models, it showed superior calibration, with a Brier score of 0.090 (95% CI, 0.063–0.119). DCA further indicated that, the LASSO-LR model provided a higher net benefit than both the “treat-all” and “treat-none” strategies across a wide range of threshold probabilities (approximately 0.05–0.45) supporting its potential clinical utility. The overall performance of each model in the test set is summarized in Figure 4 and Table 2.

**Table 2:** Performance comparisons of seven machine learning methods.

Model	AUROC (95% CI)	Accuracy	Precision	Sensitivity	Specificity	*F*_1_-score	Kappa	Youden’s *J*	PPV	NPV
Logistic	0.879 (0.814, 0.935)	0.891	0.724	0.583	0.954	0.646	0.583	0.538	0.724	0.918
DT	0.835 (0.756, 0.911)	0.820	0.480	0.667	0.851	0.558	0.449	0.518	0.480	0.925
RF	0.890 (0.825, 0.940)	0.853	0.549	0.778	0.869	0.644	0.555	0.646	0.549	0.950
XGBoost	0.893 (0.829, 0.941)	0.834	0.511	0.667	0.869	0.578	0.477	0.535	0.511	0.927
LightGBM	0.880 (0.805, 0.936)	0.867	0.595	0.694	0.903	0.641	0.560	0.597	0.595	0.935
SVM	0.868 (0.799, 0.924)	0.815	0.473	0.722	0.834	0.571	0.460	0.557	0.473	0.936
ANN	0.882 (0.822, 0.932)	0.820	0.481	0.722	0.840	0.578	0.469	0.562	0.481	0.936

### 3.5 Nomogram for predicting reintubation within 48 h in mechanically ventilated patients

**Figure 5:**
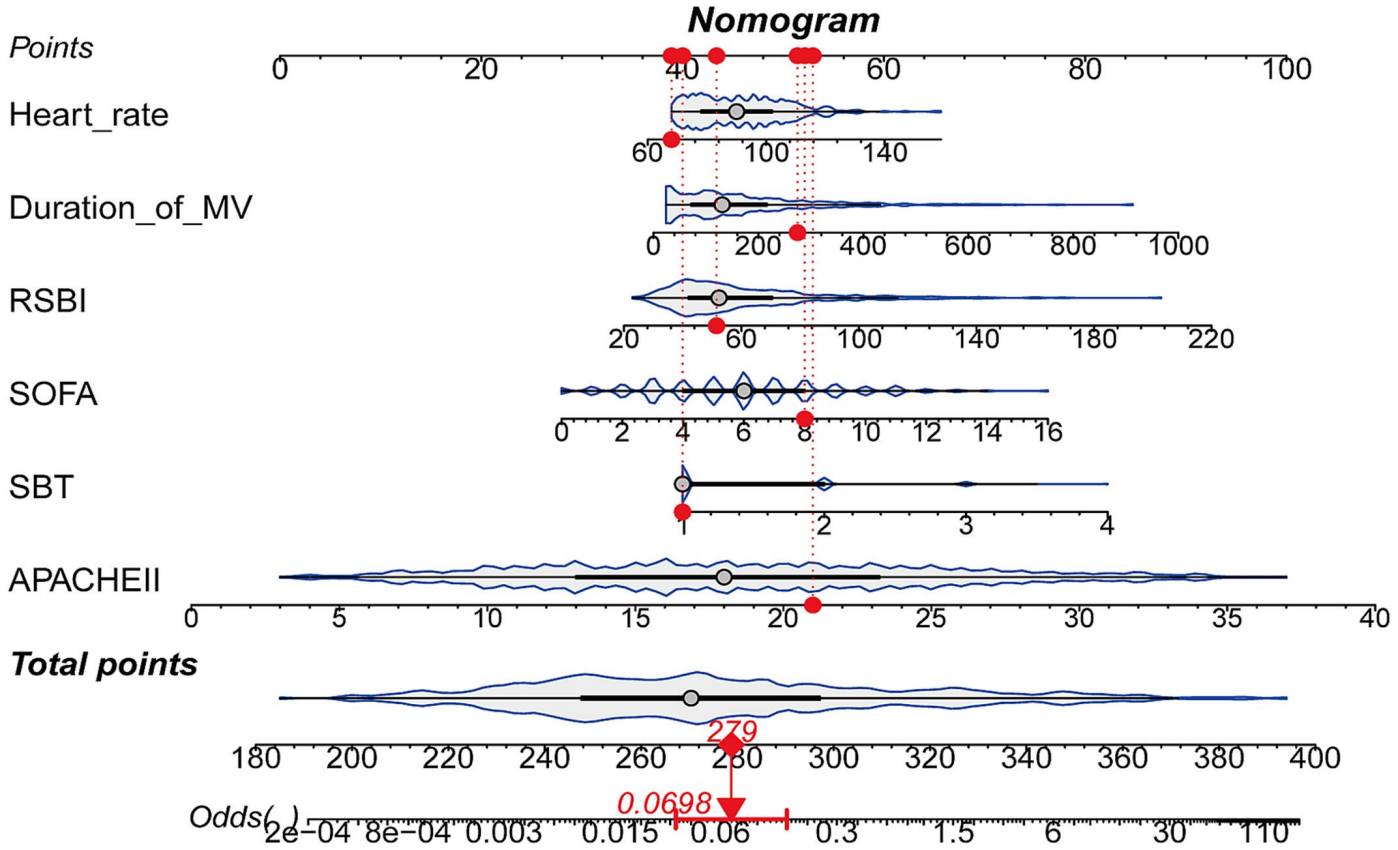
Nomogram for predicting re-intubation risk within 48 h after extubation in mechanically ventilated patients.

**Figure 6:**
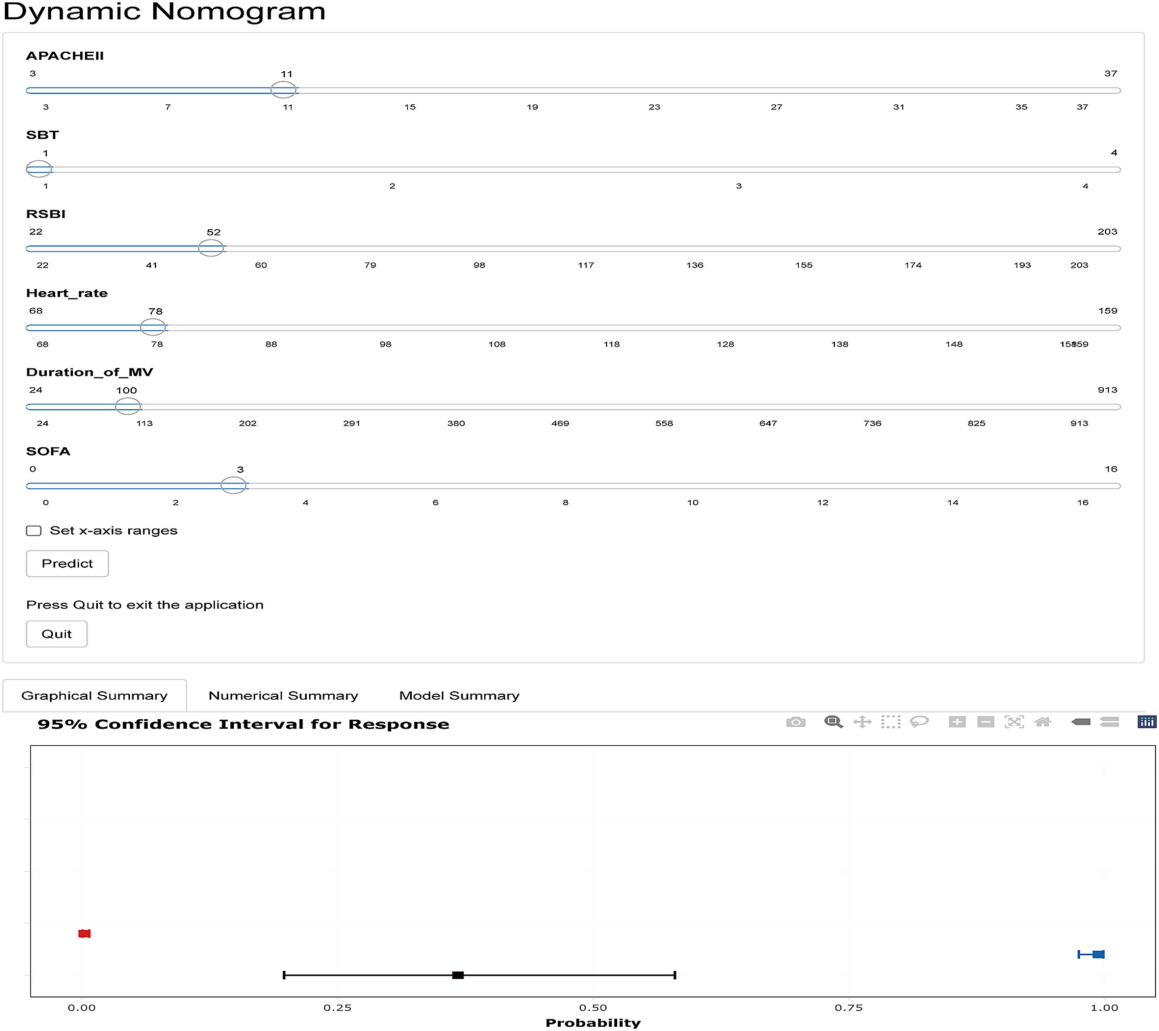
Dynamic nomogram for predicting re-intubation risk within 48 h after extubation in mechanically ventilated patients.

As described above, LASSO-LR was selected as the optimal model. It incorporated six predictors: RSBI, APACHE II score, duration of mechanical ventilation, heart rate, SOFA score, and the number of SBT attempts. Based on the multivariable logistic regression analysis, a logistic regression equation was established. The regression coefficients for the number of SBTs, RSBI, APACHE II score, SOFA score, heart rate, and duration of mechanical ventilation were 0.938, 0.019, 0.196, 0.201, 0.020, and 0.003, respectively, with an intercept of −12.615. The final model was expressed as follows: Logit(P) = −12.615 + 0.938 × number of SBTs + 0.019 × RSBI + 0.196 × APACHE II + 0.201 × SOFA + 0.020 × heart rate + 0.003 × duration of mechanical ventilation. A nomogram was subsequently constructed based on this equation. The model was further visualized as a static nomogram (Figure 5) and implemented as a web-based interactive tool.^1^ Clinicians can conveniently access this tool via mobile or desktop devices to support extubation decision-making in routine practice (Figure 6).

### 3.6 Nomogram validation

**Figure 7:**
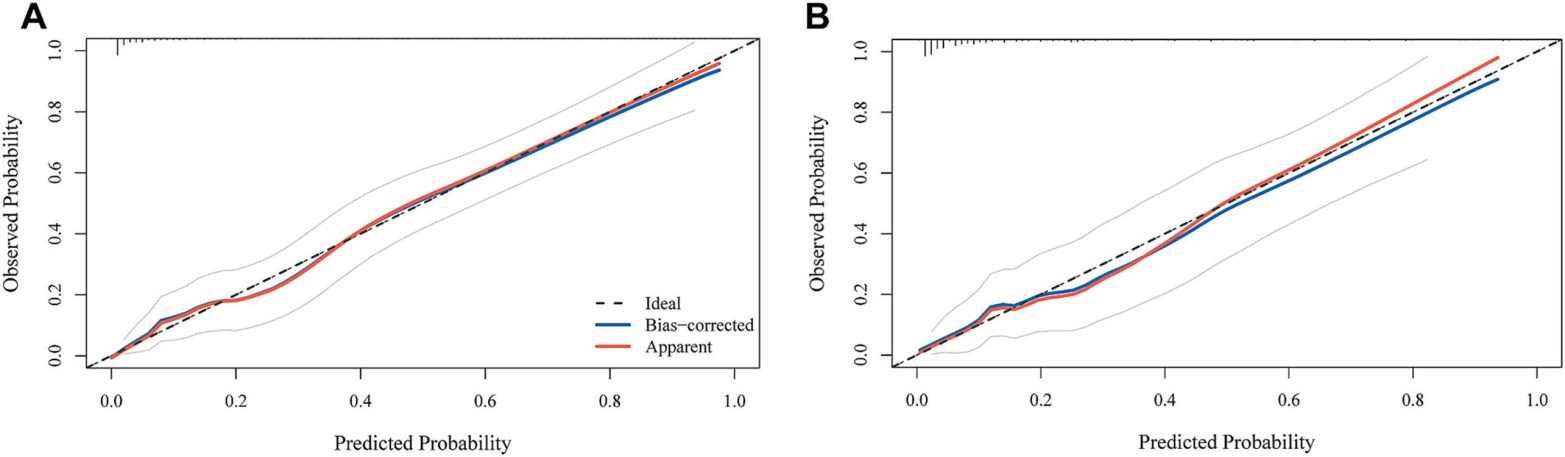
**(A)** The results of the calibration curve analysis in the training set. **(B)** The results of the calibration curve analysis in the validation set.

**Figure 8:**
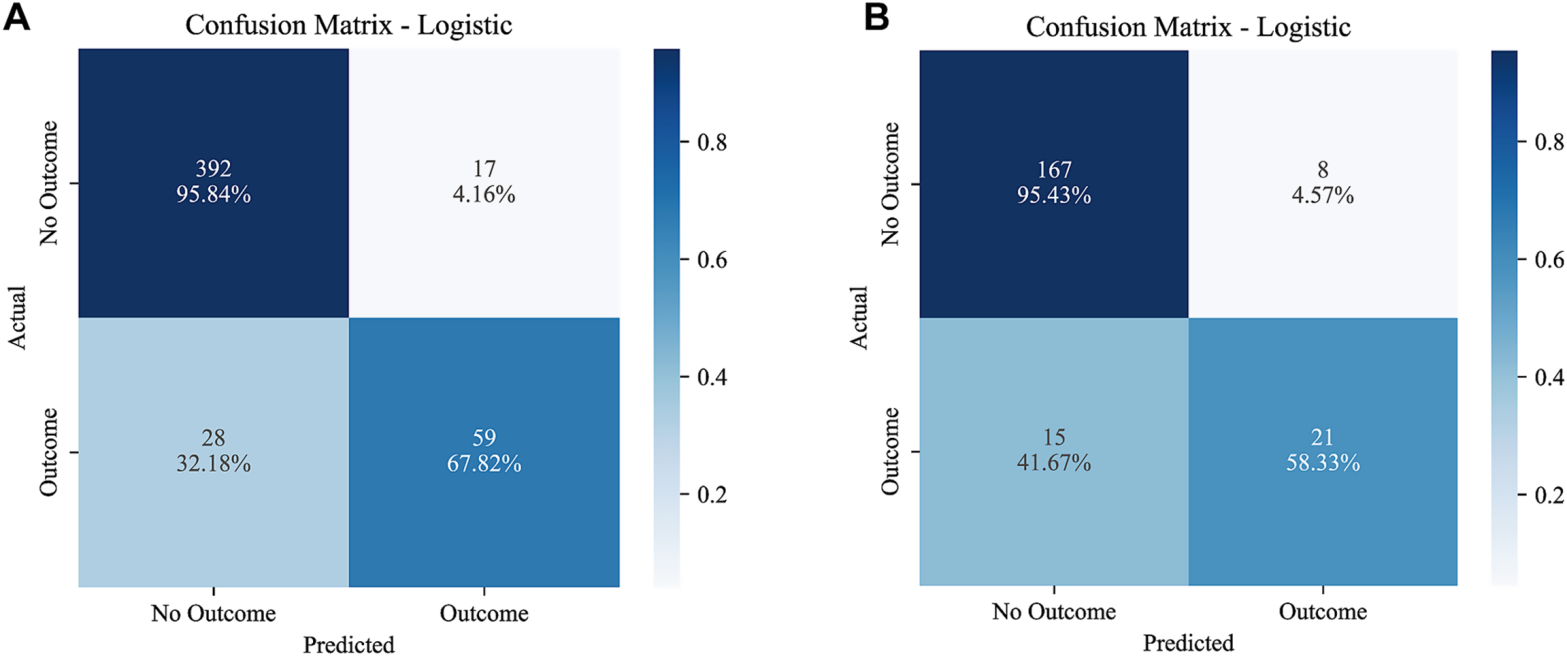
Confusion matrix of the logistic regression model: **(A)** in the training set and **(B)** in the test set.

To further assess predictive performance, calibration curves and confusion matrices were generated for both the training and test sets. The calibration curves closely followed the 45° reference line, indicating good agreement between predicted and observed risks (Figure 7). The Hosmer–Lemeshow goodness-of-fit test showed no significant discrepancy between predicted probabilities and observed event rates (training set: *χ*^2^ = 5.3954, *p* = 0.7146; test set: *χ*^2^ = 5.0112, *p* = 0.7564). The confusion matrices also suggested high specificity in both the training and test cohorts (Figure 8).

### 3.7 Comparison of the nomogram model with model based on SBT and RSBI

We incorporated two commonly used clinical extubation indicators, SBT and RSBI, into a logistic regression model and compared its performance with that of the six-variable prediction model developed in this study. The six-variable model showed better discrimination (AUROC), calibration (calibration curves), and clinical utility as assessed by decision curve analysis (DCA). Detailed results are provided in Supplementary Figure S1.

## 4 Discussion

### 4.1 Current status of reintubation within 48 h after extubation in mechanically ventilated patients

In ICU, approximately 10–15% of patients deemed ready for liberation from mechanical ventilation experience extubation failure and require reintubation; among those classified as high risk, this proportion can exceed 20% (22). Notably, reintubation should not be viewed solely as a consequence of severe illness; it has also been shown to be independently associated with adverse outcomes, including higher in-hospital mortality, prolonged duration of mechanical ventilation, and longer ICU length of stay (23). In the present study of 707 mechanically ventilated patients, we observed significant differences between the reintubation and non-reintubation groups in in-hospital mortality and ICU length of stay (*p* < 0.001), further supporting the close association between reintubation and poor prognosis.

### 4.2 Factors associated with reintubation within 48 h after extubation in mechanically ventilated patients

Reintubation within 48 h after extubation in mechanically ventilated patients is typically driven by the interplay of multiple factors. In this study, feature selection using RF-RFE and LASSO regression identified six key predictors of reintubation: RSBI, APACHE II score, duration of mechanical ventilation, heart rate, SOFA score, and the number of SBTs. Prolonged mechanical ventilation may impair the mucociliary clearance system, weaken airway defense, and is associated with airway injury, increased secretions, respiratory muscle weakness, and reduced diaphragmatic dysfunction, thereby increasing the risk of extubation failure (24–27), which is consistent with our findings. Engidaw et al. (28) further reported that, compared with patients ventilated for a shorter duration, those with prolonged ventilation (≥10 days) had an approximately 4.67-fold higher risk of extubation failure. Ventilation duration may reflect illness severity, but it can also be considered a potentially modifiable exposure; therefore, extubation readiness should be assessed as early as feasible to minimize ventilation-related complications.

The SBT provides an objective assessment of spontaneous breathing capacity and informs integrated decision-making before extubation; its failure criteria generally include both subjective signs/symptoms and objective physiological indicators (29). A higher number of SBTs often indicates insufficient respiratory reserve during weaning or incomplete clinical recovery. Even when patients ultimately pass the SBT and undergo planned extubation, they may still decompensate shortly thereafter due to marginal respiratory muscle endurance, inadequate airway protection, or increased cardiopulmonary workload, necessitating reintubation (30–33). Some studies suggest that, for patients who require multiple SBTs before passing, pressure support ventilation with a higher inspiratory pressure (5–8 cmH₂O) during the initial SBT, coupled with closer monitoring after extubation, may be beneficial; in appropriately selected patients, prophylactic noninvasive ventilation strategies may also be considered to reduce reintubation risk (34).

With respect to acute illness severity, patients who required reintubation differed significantly from those with successful extubation, with higher SOFA and APACHE II scores in the reintubation group. As established indices of disease severity (35, 36), elevated SOFA and APACHE II scores generally reflect a greater degree of multi-organ dysfunction and diminished physiological reserve (37, 38). Prior studies defining outcomes as reintubation within 48 h after extubation or extubation failure have similarly shown that more severe organ dysfunction (e.g., higher SOFA/APACHE II) is associated with an increased risk of early extubation failure, thereby providing clinical support for the inference that greater illness severity translates into a higher likelihood of early reintubation (28, 39).

An elevated RSBI reflects a rapid, shallow breathing pattern, indicating borderline ventilatory capacity during weaning; this pattern is commonly linked to reduced respiratory muscle endurance, lower ventilatory efficiency, and increased work of breathing (7). Among patients who pass the SBT, higher RSBI has been associated with extubation failure and an increased likelihood of reintubation (40, 41).

Heart rate prior to extubation was defined as the value recorded 30 min before extubation, reflecting the patient’s immediate physiological status at the time of the procedure. In routine weaning practice, cardiovascular stability is generally considered a prerequisite for SBT initiation and tolerance, and heart rate thresholds and/or an elevated baseline heart rate are often used as objective warning signals for SBT failure (29, 42). Some ICU protocols also incorporate the absence of overt tachycardia as part of extubation criteria (43). Observational studies have consistently suggested that abnormal heart rate remains an independent risk factor for extubation failure in multivariable models (44). Taken together, an increased pre-extubation heart rate in our cohort may reflect heightened sympathetic activation, limited cardiopulmonary reserve, or unrecognized contributors such as infection/inflammatory stress, pain, agitation, or increased work of breathing (45). Accordingly, it may function more as a bedside “early warning” marker of extubation failure risk. Because this measure captures real-time bedside physiology, using it for risk stratification at the extubation time point and to guide intensified monitoring or supportive strategies in high-risk patients may have practical clinical value.

### 4.3 Clinical implications of predicting reintubation risk after extubation

Early reintubation after extubation often follows a dynamic clinical trajectory. It may begin with impaired secretion clearance or upper airway obstruction and then progress to respiratory muscle fatigue, atelectasis, pulmonary edema, and even progressive respiratory failure (23, 46). This time-varying, multifactorial evolution underscores the importance of accurate risk assessment prior to extubation, as it can directly influence the timing of extubation and subsequent management strategies. Objective estimation of reintubation risk may also support rational allocation of ICU resources and streamline decision-making workflows. Accordingly, the web-based dynamic nomogram developed in this study is based on bedside variables that are readily available before extubation. When interpreted alongside DCA, the model allows quantitative risk stratification at the time of extubation and may assist clinicians in tailoring post-extubation monitoring intensity and respiratory support strategies. For patients classified as high risk (predicted probability ≥25%), closer surveillance of vital signs and respiratory status is advisable. Multidisciplinary discussion may be appropriate to evaluate the timing of extubation, and airway management equipment should be readily available at the bedside in anticipation of potential clinical deterioration. For those at intermediate risk (predicted probability 15–25%), prophylactic respiratory support after extubation can be considered in light of current evidence. This may include noninvasive ventilation (NIV) or high-flow nasal cannula (HFNC), both of which have been shown to reduce the incidence of post-extubation respiratory failure and reintubation in selected populations (47–50). By contrast, patients identified as low risk (predicted probability <15%) may be managed with standard monitoring while avoiding unnecessary interventions. Such an approach may enhance nursing efficiency and promote more appropriate use of healthcare resources without compromising patient safety.

### 4.4 Strengths and limitations

In this retrospective study, we analyzed clinical data from 707 mechanically ventilated patients and developed, compared, and visualized multiple machine-learning models using nomograms, followed by a systematic evaluation of model performance. Nomograms have become increasingly common in recent years as a practical format for translating prediction models into bedside tools. Based on the overlap between LASSO and RF-RFE, we identified six key predictors and constructed both a static nomogram and a web-based dynamic nomogram (see text footnote 1). After external testing within the held-out test set, the nomogram demonstrated good discrimination and calibration and provided a measurable net clinical benefit, suggesting reasonable stability and clinical potential. The tool is intuitive and straightforward to use, allowing clinicians to perform individualized risk assessment at a critical extubation decision point using readily available bedside features. This may help reduce “trial-and-error” extubation attempts and associated reintubation risk, while also offering a reference for tailoring monitoring intensity and allocating resources, thereby improving overall workflow efficiency and potentially reducing costs. In addition, relatively strict inclusion and exclusion criteria were applied, supporting data quality and yielding an adequate sample size for model development.

Several limitations should be acknowledged. First, the retrospective design makes residual selection and information bias difficult to fully exclude. Second, although SMOTE can mitigate class imbalance, synthetic oversampling may introduce modest distributional shifts; future work could incorporate sensitivity analyses using alternative approaches such as class-weighted loss functions or focal loss to further assess robustness. Third, the relatively low overall incidence of reintubation in this cohort may affect the precision of effect estimates and the stability of confidence intervals. Fourth, this was a single-center retrospective study with internal validation only. Because both training and test cohorts were derived from the same institutional population and clinical practice pattern, performance estimates may be subject to optimism bias. Temporal validation and external validation in independent institutions are required to assess generalizability before clinical implementation.

## 5 Conclusion

Our study selected the optimal model and visualized it as a static and dynamic nomogram. This exploratory model demonstrates the potential of integrating routinely available bedside variables for early risk stratification of reintubation within 48 h after extubation. However, given the single-center retrospective design and lack of external validation, these findings should be considered hypothesis-generating. Further multicenter validation and prospective studies are required before clinical implementation.

## Data Availability

The datasets generated and/or analyzed during the current study are not publicly available due to institutional regulations and patient privacy restrictions. De-identified data may be made available from the corresponding author upon reasonable request and subject to approval by the institutional ethics committee and relevant data-use agreements. Requests to access these datasets should be directed to dixw@jzmu.edu.cn.

## Footnotes

1
https://predict-for-reintubation-within-48-hours.shinyapps.io/dynnomapp/

## Supplementary material

SUPPLEMENTARY FIGURE S1 **(A)** ROC curve of the nomogram, **(B)** DCA curve of the nomogram, **(C)** calibration plot of the nomogram, **(D)** confusion matrix of the nomogram, **(E)** ROC curve of the model based on SBT and RSBI, **(F)** DCA curve of the model based on SBT and RSBI, **(G)** calibration plot of the model based on SBT and RSBI, **(H)** confusion matrix of the model based on SBT and RSBI.
