# Supplementary figures and images for: Construction and validation of a machine learning-based prediction model for 48-hour reintubation risk in mechanically ventilated patients
Source: Front Med (Lausanne). 2026 Mar 19;13:1788254. doi: 10.3389/fmed.2026.1788254 (PMC13044105; doi:10.3389/fmed.2026.1788254)

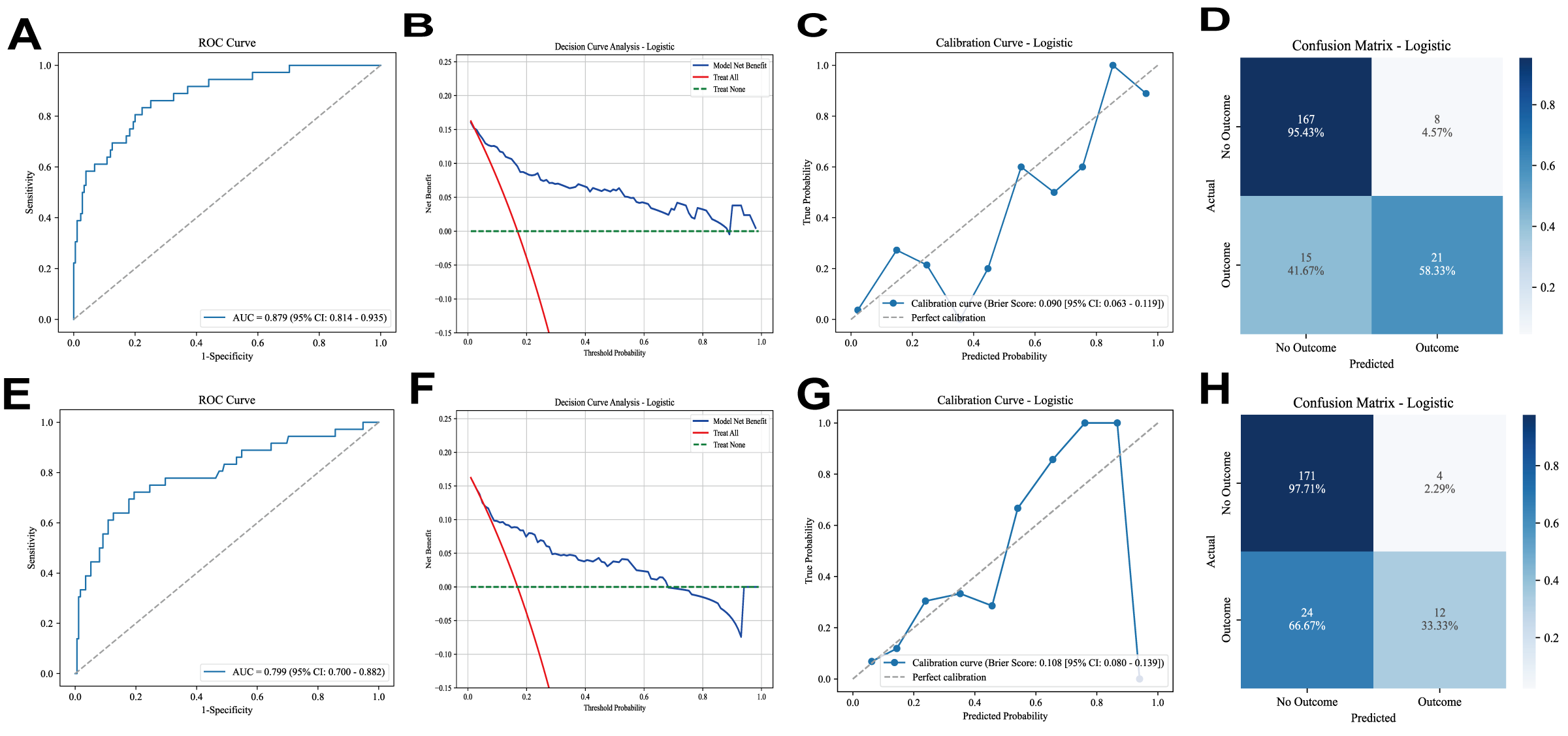

Supplement: SUPPLEMENTARY FIGURE S1 — (A) ROC curve of the nomogram, (B) DCA curve of the nomogram, (C) calibration plot of the nomogram, (D) confusion matrix of the nomogram, (E) ROC curve of the model based on SBT and RSBI, (F) DCA curve of the model based on SBT and RSBI, (G) calibration plot of the model based on SBT and RSBI, (H) confusion matrix of the model based on SBT and RSBI. [file Image_1.tif]
